# FastSCODE: an accelerated SCODE algorithm for inferring gene regulatory networks on manycore processors

**DOI:** 10.1093/bioinformatics/btaf624

**Published:** 2025-11-14

**Authors:** Rakbin Sung, Seongmi Woo, Dongmin Shin, Junil Kim, Daewon Lee

**Affiliations:** Department of Applied Art and Technology, College of Art and Technology, Chung-Ang University, Anseong 17546, Republic of Korea; Department of Applied Art and Technology, College of Art and Technology, Chung-Ang University, Anseong 17546, Republic of Korea; Department of Applied Art and Technology, College of Art and Technology, Chung-Ang University, Anseong 17546, Republic of Korea; School of Systems Biomedical Science, Soongsil University, Seoul 06978, Republic of Korea; Department of Bioinformatics, Soongsil University, Seoul 06978, Republic of Korea; Department of Applied Art and Technology, College of Art and Technology, Chung-Ang University, Anseong 17546, Republic of Korea; School of Art and Technology, College of Art and Technology, Chung-Ang University, Anseong 17546, Republic of Korea

## Abstract

**Summary:**

SCODE reconstructs gene regulatory networks from single-cell RNA sequencing (scRNA-seq) data using an ordinary differential equation (ODE) model, and has been successfully applied to a wide range of scRNA-seq datasets, including mouse, human, and plant cells. However, its computational performance is limited when processing large datasets due to its sequential execution flow and repeated optimization loops. To overcome this limitation, we have developed FastSCODE, a batch computing version of the SCODE algorithm optimized for acceleration on manycore processors such as GPUs. FastSCODE performs batch computation on multiple gene expression profiles and optimizes the parameters of a linear ODE model using manycore computing. Compared to the original implementation, FastSCODE achieves up to 6000× improvement in performance (from about one month to 10 min) on the CeNGEN scRNA-seq dataset when using multiple GPUs.

**Availability and implementation:**

FastSCODE is publicly available on GitHub at https://github.com/cxinsys/fastscode.

## 1 Introduction

The expression values of genes can be tracked through RNA sequencing. The process of bulk RNA sequencing involves the calculation of the mean expression value of a given gene across multiple cells. This results in low resolution for expression dynamics from each individual cell. Single-cell RNA sequencing (scRNA-seq) acquires gene expressions from individual cells, with the objective of expanding our understanding of cellular dynamics. Applying computational algorithms to scRNA-seq data enables the inference of gene regulatory networks (GRNs), providing a profound understanding of the intricate mechanisms underlying complex biological phenomena (Pratapa *et al.* 2020, Kim *et al.* 2024). Consequently, the advent of scRNA-seq has led to the accumulation of large-scale datasets, thereby increasing the demand for highly efficient and scalable algorithms and software for big data analysis (Eisenstein 2020, Andrews *et al.* 2021, Granja *et al.* 2021). For example, GRNBoost2 is an accelerated version of GENIE3 that utilizes parallel processing with multiple CPUs (Moerman *et al.* 2019), while FastTENET achieves up to a 973-fold speedup over the original TENET by using GPU-based manycore computing (Sung *et al.* 2024).

SCODE is a GRN inference algorithm that models gene expression dynamics using a linear ODE framework and estimates regulatory influences through linear regression optimization (Matsumoto *et al.* 2017). The resulting score matrix quantitatively represents the strength of regulatory relationships between transcription factors and their target genes. SCODE has demonstrated its effectiveness in identifying key regulators through GRN inference across a variety of datasets, including mouse embryonal carcinoma (EC) cells, mouse embryonic fibroblasts (MEFs), and human EC cells (Matsumoto *et al.* 2017). Its application has extended to reconstructing GRNs from sequencing data obtained from plant tissues such as root, pericarp, and stem cells (Denyer *et al.* 2019, Zhang *et al.* 2021, Li *et al.* 2024), as well as from immune cell populations (Yossef *et al.* 2023).

As a representative algorithm based on a linear ODE model, SCODE is often used as a benchmark in comparative evaluations of newly developed GRN inference methods (Pratapa *et al.* 2020, Ramachandran *et al.* 2020, Shu *et al.* 2021, Su *et al.* 2022, Wang *et al.* 2022, Zinati *et al.* 2024, Chen *et al.* 2025). However, its original implementation suffers from limited computational efficiency, particularly when applied to large-scale datasets. The sequential processing of gene expression profiles and the iterative nature of ODE-based optimization, which involves multiple nested loops, lead to substantial performance degradation as the number of genes increases. To overcome the computational limitations of the original SCODE implementation, we have developed “FastSCODE,” which is accelerated through parallel processing on manycore processors. FastSCODE reduces the number of optimization loops by performing batch computation across multiple gene expression profiles and corresponding parameter vectors of the linear ODE model. It is specifically designed to support high performance manycore architectures such as GPUs, TPUs, and NPUs, which are well suited for large-scale batch operations. In FastSCODE, users can select an acceleration framework for manycore computing, such as NumPy (Harris *et al.* 2020), PyTorch (Paszke *et al.* 2019), CuPy (Okuta *et al.* 2017), TensorFlow (Abadi *et al.* 2015), or JAX (Bradbury *et al.* 2018), all of which are supported through a unified array computing interface. As a result, FastSCODE achieved more than an 800-fold speedup compared to the original SCODE implementation when executed on four NVIDIA RTX 4090 GPUs.

## 2 Methods

### 2.1 Basic concept

The SCODE algorithm quantifies regulatory relationships among genes by computing a score matrix from gene expression data. The observed gene expression data can be represented as $X\in R^{G\times C}$, where *G* and *C* denote the number of genes and cells, respectively. Equation (1) shows how SCODE models gene expression dynamics with a linear ODE model.


(1) $$\begin{array}{c} \frac{dx}{dt}=\mathrm{Ax}, \end{array}$$

where $x\in R^{G}$ is a vector of the gene expression at the time *t*, and $A\in R^{G\times G}$ denotes the score matrix that indicates the strength of the relationship between genes in GRNs. However, the direct optimization of A has a time complexity of $O(CG^{3})$, leading to computationally expensive for large-scale datasets. For reducing the computational complexity, SCODE introduces an additional linear ODE equation with a low-dimensional latent vector z, as shown in Equation (2).


(2) $$\begin{array}{c} dz=\mathrm{Bz}dt,\\ x=\mathrm{Wz}, \end{array}$$

where $z\in R^{D}$ (D≪G) represents the latent vector that captures the underlying gene expression dynamics, and $B\in R^{D\times D}$ defines the linear dynamics of z in the latent space. The matrix $W\in R^{G\times D}$ is a linear mapping from the latent space to the gene expression vector. Given that W satisfies x=Wz, the ODE of x can be derived according to Equation (3).


(3) $$\begin{array}{c} dx\approx \mathrm{WB}W^{+}xdt, \end{array}$$

where $W^{+}$ denotes the Moore-Penrose pseudoinverse of W. The score matrix A can be approximated by $A\approx \mathrm{WB}W^{+}$ according to Equations (1) and (3). SCODE optimizes the matrix B through Monte Carlo sampling. In the first step of optimization, the diagonal elements of B, denoted by the vector $b\in R^{D}$, are initialized with values uniformly sampled from the range [$b_{\text{min}},b_{\text{max}}$]. Following the sampling of b, the latent representation matrix Z is constructed by computing the general solution $Z_{i,c}=e^{b_{i}t_{c}}$, where $b_{i}$ is the *i*-th diagonal element of B and $t_{c}$ denotes the *c*-th pseudotime point. Subsequently, W is derived from Z according to $W^{⊤}={\left(ZZ^{⊤}\right)}^{-1}ZX^{⊤}$ based on the relationship $X^{⊤}=Z^{⊤}W^{⊤}$. SCODE calculates the residual sum of squares (RSS) between X and WZ. During the optimization loop, one element of b is randomly sampled and updated in each iteration to minimize the RSS. Finally, the score matrix A is estimated as $A\approx WB_{\mathrm{best}}W^{+}$, where W is computed using the matrix $B_{\mathrm{best}}$ that achieves the minimum RSS.

Although the original implementation of SCODE incorporates several techniques to mitigate computational complexity, it still requires a substantial amount of execution time due to independent computations for each gene and repeated optimization steps. It is evident that the execution time of SCODE increases dramatically with the number of genes and optimization iterations, making it computationally prohibitive for large-scale datasets.

### 2.2 Batch computing for reducing repetition

**Figure 1.**
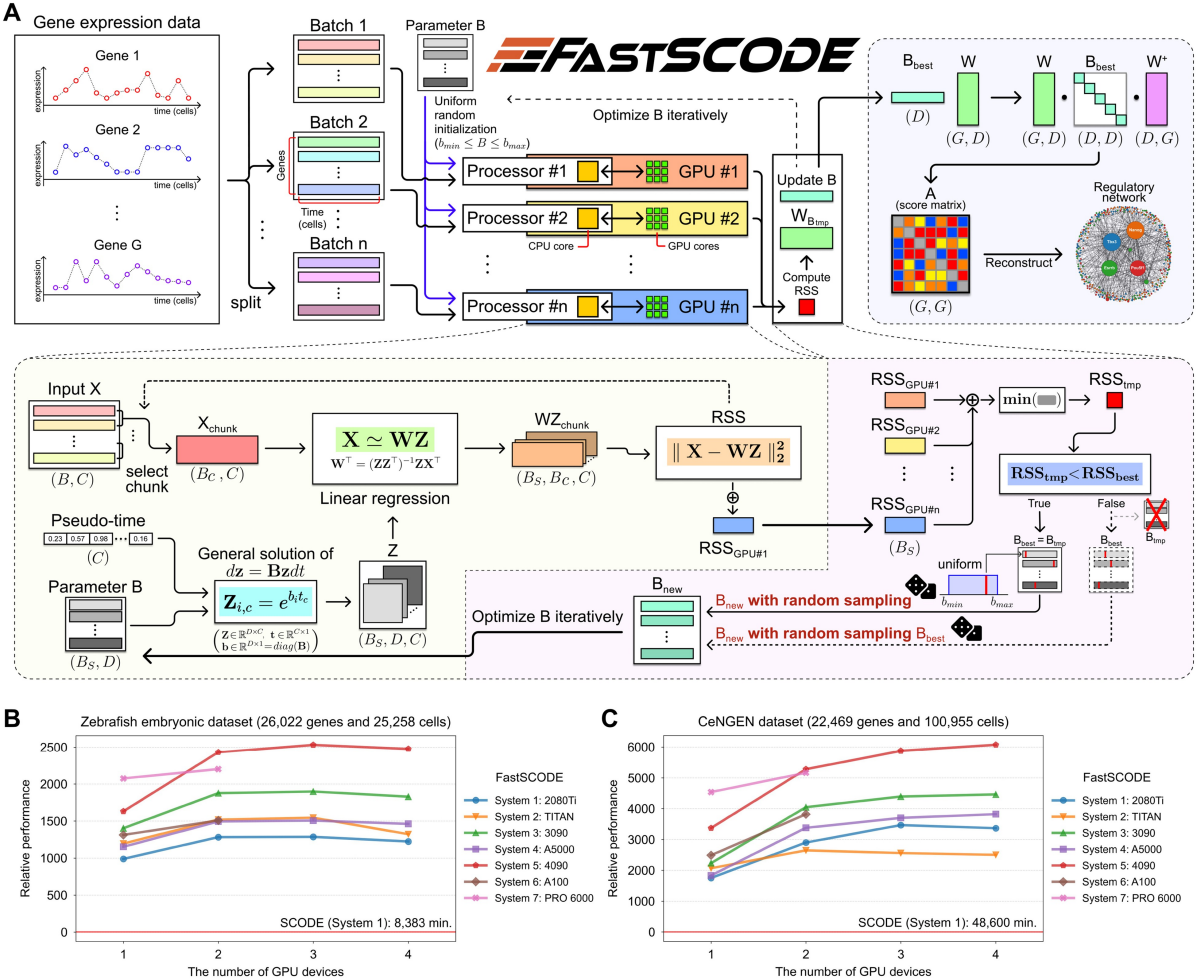
Overview of FastSCODE and performance analysis. (A) Schematic overview of FastSCODE algorithm. (B and C) Relative performance of FastSCODE on the (B) zebrafish embryonic dataset, and (C) CeNGEN dataset across multiple computing systems. All baseline experiments of the original SCODE were conducted using 32 CPU cores of Intel Xeon Silver 4214R on System 1. Refer to Table 1, available as supplementary data at *Bioinformatics* online for details on system configurations.

FastSCODE introduces batch array computing when solving linear regression to minimize the repeated computations for each gene. A subset of gene expressions is uploaded to a manycore processor, according to batch size *B* of FastSCODE. Depending on the chunk size $B_{c}$, FastSCODE computes the linear transformation on multiple gene expressions (Fig. 1A). If sufficient computational resources are available, computations across all genes can be executed in a single batch, eliminating the memory transfer overhead between the CPU and manycore processor. Furthermore, FastSCODE expands B into a $B_{S}\times D$ matrix for the parallel computation of $B_{S}$ RSS values (Fig. 1A), significantly reducing the number of required optimization iterations while preserving the original random sampling strategy.

### 2.3 Parallel processing on manycore processors

In FastSCODE, scRNA-seq datasets are partitioned into batches according to the available manycore processors. Multiple worker processes are launched, each assigned to a specific manycore processor to perform linear transformations and RSS computations on designated data chunks. Users can configure the list of accessible manycore processors and specify the batch and chunk size for each processing unit. After computation, the RSS results from all batches are aggregated into a single array in CPU memory managed by the main process (Fig. 1A). Numerical operations involving large arrays can benefit from substantial speedup when executed on specialized manycore processors such as GPUs, NPUs, and TPUs. FastSCODE supports a variety of acceleration frameworks such as CuPy, JAX, TensorFlow, and PyTorch.

### 2.4 Experimental setup

To evaluate the performance of FastSCODE in comparison to SCODE, we conducted a series of experiments using four scRNA-seq datasets. The mouse embryonic stem cell (mESC) neural differentiation (Setty *et al.* 2016, Tuck *et al.* 2018) and the skin cancer (Gaydosik *et al.* 2020, Ji *et al.* 2020, Kfoury *et al.* 2021) datasets were selected, as they consist of fewer than several thousand genes and cells. Furthermore, two large-scale datasets were used: the zebrafish embryonic (Wagner *et al.* 2018) and the complete gene expression map of an entire nervous system (CeNGEN) (Hammarlund *et al.* 2018) datasets.

We evaluated the performance of FastSCODE across seven distinct computing systems (Table 1, available as supplementary data at *Bioinformatics* online). The execution time of SCODE on System 1 was used as the reference baseline for all comparisons. FastSCODE was tested using up to 4 GPUs, while for Systems 6 and 7, it utilized up to 2 GPUs, depending on hardware configuration of the system. The batch size was set as the number of genes divided by the number of GPUs, while the chunk size was dynamically adjusted by the algorithm. Relative performance was calculated by dividing the execution time of SCODE by that of FastSCODE. Additionally, we analyzed the runtime breakdown by measuring the resource utilization of FastSCODE and SCODE.

## 3 Results

### 3.1 Computation speed improvement

On the zebrafish dataset, FastSCODE achieved up to a 2532-fold speedup when utilizing three NVIDIA RTX 4090 GPUs (Fig. 1B). Even greater acceleration was observed for the CeNGEN dataset, where FastSCODE achieved >6000-fold speedup using four 4090 GPUs (Fig. 1C). The actual execution time was reduced from 8383 to 3.3 min for the zebrafish dataset and from 48 600 to 8 min for the CeNGEN dataset, respectively (Fig. 1, available as supplementary data at *Bioinformatics* online). On the zebrafish dataset, FastSCODE achieved its maximum performance across all computing systems when utilizing two or three GPUs (Fig. 1B). However, on the CeNGEN dataset, FastSCODE achieved its maximum performance across most computing systems when utilizing three or four GPUs (Fig. 1C).

FastSCODE achieved speed improvements of up to 505-fold on the mESC dataset and 1050-fold on the skin cancer dataset when utilizing the NVIDIA RTX 4090 GPU and PRO 6000, respectively (Fig. 2, available as supplementary data at *Bioinformatics* online). Both datasets were small enough for FastSCODE to compute the entire dataset on a single GPU at once. However, despite increasing the number of GPU devices, the total execution time unexpectedly increased (Figs 1 and 2, available as supplementary data at *Bioinformatics* online).

### 3.2 Resource utilization analysis

Since increasing the number of GPU devices did not necessarily result in performance improvement, we measured and analyzed both the time spent on computing tasks and the temporal variation of CPU and GPU utilization (%) during runtime (Figs 3–5, available as supplementary data at *Bioinformatics* online). These results reveal a distinct trade-off between CPU-to-GPU memory transfer and GPU computation. As the number of devices increases, the proportion of time spent on CPU-to-GPU data transfer grows markedly, indicating a substantial communication overhead introduced by multi-device parallelization. Consequently, effective multi-GPU acceleration can be achieved only when the computational workload is sufficiently large to dominate the data-transfer overhead, as observed in large-scale datasets such as zebrafish and CeNGEN.

### 3.3 Detailed performance analysis

We examined how the batch size, one of the most critical hyperparameters, affects the runtime performance of FastSCODE (Figs 6 and 7, available as supplementary data at *Bioinformatics* online). When the batch size is set to the entire dataset, the overhead from data transfer was reduced, leading to a substantial enhancement in computation speed. Increasing the batch size for matrix B while reducing the number of optimization iterations decreased execution time, highlighting the importance of reducing repeated sampling and evaluation steps in the optimization process. We also compared manycore acceleration frameworks (Figs 8 and 9, available as supplementary data at *Bioinformatics* online). For larger datasets, parallelization effectively amortizes computational overhead across GPUs, narrowing the performance differences among frameworks. In contrast, for smaller datasets, data transfer overhead dominates and amplifies performance gaps, particularly in frameworks showing lower parallel efficiency. The consistency between the results of FastSCODE and the original SCODE was also evaluated (Fig. 10, available as supplementary data at *Bioinformatics* online).

## 4 Conclusion

FastSCODE is an accelerated implementation of SCODE that uses parallel processing on manycore processors to enhance computational efficiency. FastSCODE was designed to accelerate SCODE by introducing batch array computing into the linear regression phase, targeting the elimination of repetitive computations for each gene. Our experiments demonstrate that FastSCODE achieves substantial scalability and runtime improvements in the GRN inference from large-scale scRNA-seq datasets. FastSCODE eliminates the major computational bottlenecks of SCODE, transforming it into a highly scalable and efficient algorithm. Given its performance and scalability, FastSCODE offers a practical and efficient solution for GRN inference in bioinformatics and biomedical research.

## Supplementary Material

btaf624_Supplementary_Data

## Data Availability

FastSCODE with test dataset is available online for public use at https://github.com/cxinsys/fastscode
