# Supplementary material for: FastSCODE: an accelerated SCODE algorithm for inferring gene regulatory networks on manycore processors
Source: Bioinformatics. 2025 Nov 14;41(12):btaf624. doi: 10.1093/bioinformatics/btaf624 (PMC12684706; doi:10.1093/bioinformatics/btaf624)
Supplement: btaf624_Supplementary_Data [file btaf624_supplementary_data.pdf]

Supplementary Data

FastSCODE: an accelerated SCODE algorithm for inferring gene regulatory networks on manycore processors

Rakbin Sung[1], Seongmi Woo[1], Dongmin Shin[1], Junil Kim[2,3], and Daewon Lee[1,4]

[1] *Department of Applied Art and Technology, Chung-Ang University, Anseong 17546, Republic of Korea*
[2] *School of Systems Biomedical Science, Soongsil University, Seoul 06978, Republic of Korea*
[3] *Department of Bioinformatics, Soongsil University, Seoul 06978, Republic of Korea*
[4] *School of Art and Technology, Chung-Ang University, Anseong 17546, Republic of Korea*

Supplementary Notes

1 Repository

We have provided a GitHub repository to facilitate the open-source project of the FastSCODE framework. You can find the implementation in Python and helpful tutorials on how to get started with FastSCODE in the repository.

- Repository: https://github.com/cxinsys/fastscode
- Tutorials: https://github.com/cxinsys/fastscode/tree/main/tutorials

2 Overall flow of the SCODE algorithm

The sequential optimization steps of the SCODE algorithm are summarized as follows.

1. **Input**: The gene expression matrix $\mathbf{X} \in \mathbb{R}^{G \times C}$ and the pseudotime vector of length C.
2. **Initialization**: Initialize the diagonal elements of $\mathbf{B}$ by sampling random values from a uniform distribution over a range $[b_{min}, b_{max}]$.
3. **Latent representation**: Derive the latent representation $\mathbf{Z} \in \mathbb{R}^{D \times C}$ $(D \ll G)$ from the general solution of the linear ODE: $d\mathbf{z} = \mathbf{B}\mathbf{z}dt \rightarrow \mathbf{Z}_{i,c} = e^{b_i t_c}$, where b_i is the i-th diagonal element of $\mathbf{B}$ and t_c is the c-th pseudotime point.
4. **Estimate the matrix W**: Compute $\mathbf{W}^\top = (\mathbf{Z}\mathbf{Z}^\top)^{-1}\mathbf{Z}\mathbf{X}^\top$ based on the relationship $\mathbf{X} = \mathbf{W}\mathbf{Z}$ (i.e., $\mathbf{X}^\top = \mathbf{Z}^\top\mathbf{W}^\top$).
5. **Objective evaluation**: Calculate the residual sum of squares (RSS) between $\mathbf{X}$ and $\mathbf{W}\mathbf{Z}$.
6. **Update the best B**: If the current RSS is smaller than the minimum RSS obtained so far, update $\mathbf{B}_{best} = \mathbf{B}$; otherwise, retain the previous $\mathbf{B}_{best}$.
7. **Resample an element of B**: Randomly select one diagonal element of $\mathbf{B}$ and resample its value within the predefined range.
8. **Iteration**: Repeat steps 3-7 for a specified number of iterations to optimize $\mathbf{B}$.
9. **Output**: Compute the final score matrix as $\mathbf{A} = \mathbf{W}\mathbf{B}\mathbf{W}^+$ using $\mathbf{W}$ obtained from $\mathbf{B}_{best}$ (i.e., $\mathbf{W}^\top = (\mathbf{Z}_{best}\mathbf{Z}_{best}{}^\top)^{-1}\mathbf{Z}_{best}\mathbf{X}^\top$).

Supplementary Figures and Tables

Table S1. Computing system configurations for performance analysis.

System	Device	Product	Specification
System 1	CPU	Intel Xeon Silver 4214R	48 cores<br>512 GB memory
	GPU	NVIDIA RTX 2080Ti	4,352 CUDA cores<br>11 GB memory
System 2	CPU	Intel Core i9-10980XE	36 cores<br>128 GB memory
	GPU	NVIDIA TITAN RTX	4,608 CUDA cores<br>24 GB memory
System 3	CPU	AMD EPYC 7702	128 cores<br>256 GB memory
	GPU	NVIDIA RTX 3090	10,496 CUDA cores<br>24 GB memory
System 4	CPU	Intel Xeon Silver 4214R	48 cores<br>1,024 GB memory
	GPU	NVIDIA RTX A5000	8,192 CUDA cores<br>24 GB memory
System 5	CPU	AMD Ryzen Threadripper Pro 5955WX	32 cores<br>256 GB memory
	GPU	NVIDIA RTX 4090	16,384 CUDA cores<br>24 GB memory
System 6	CPU	Intel Xeon Gold 6338	16 cores<br>128 GB memory
	GPU	NVIDIA A100	6,912 CUDA cores<br>80 GB memory
System 7	CPU	Intel Core i9-10980XE	36 cores<br>256 GB memory
	GPU	NVIDIA RTX PRO 6000 Blackwell	24,064 CUDA cores<br>96 GB memory

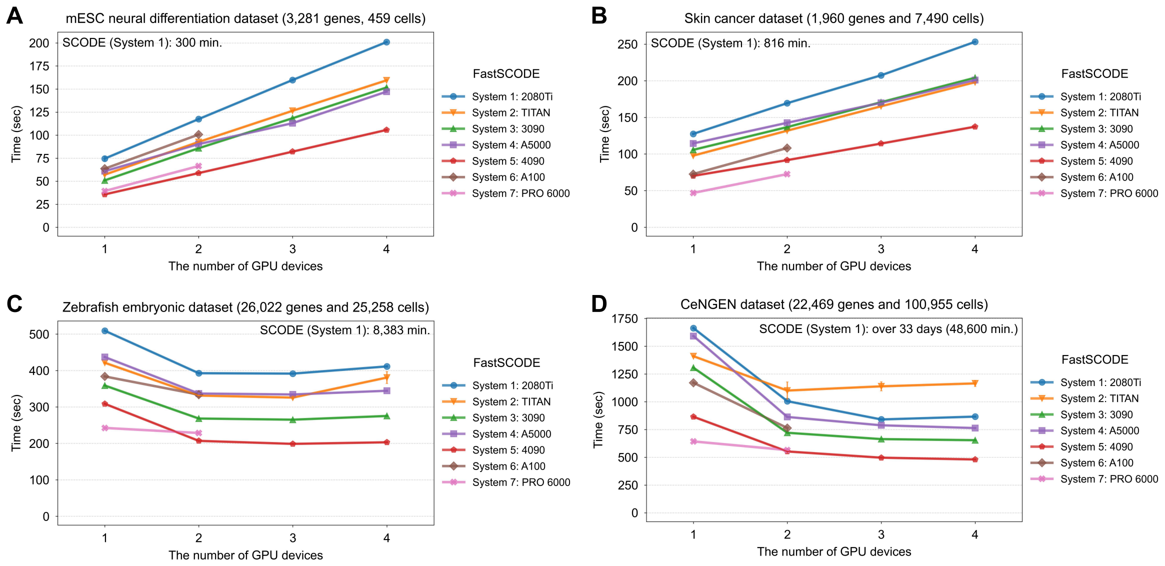

Figure S1. Execution times of FastSCODE on the **(A)** mESC dataset, **(B)** skin cancer dataset, **(C)** zebrafish embryonic dataset, and **(D)** CeNGEN dataset. System 1 (Intel Xeon Silver 4214R 2.4GHz CPU) was used for all experiments of the original SCODE.

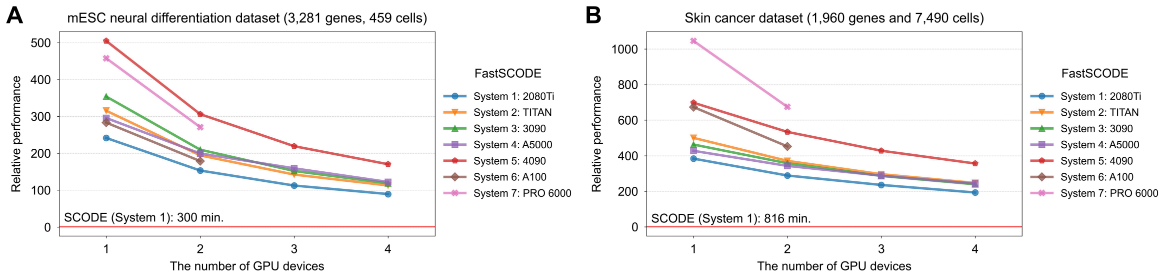

Figure S2. Relative performance of FastSCODE on the (**A**) mESC neural differentiation dataset, and (**B**) skin cancer dataset. System 1 (Intel Xeon Silver 4214R 2.4GHz CPU) was used for all experiments of the original SCODE.

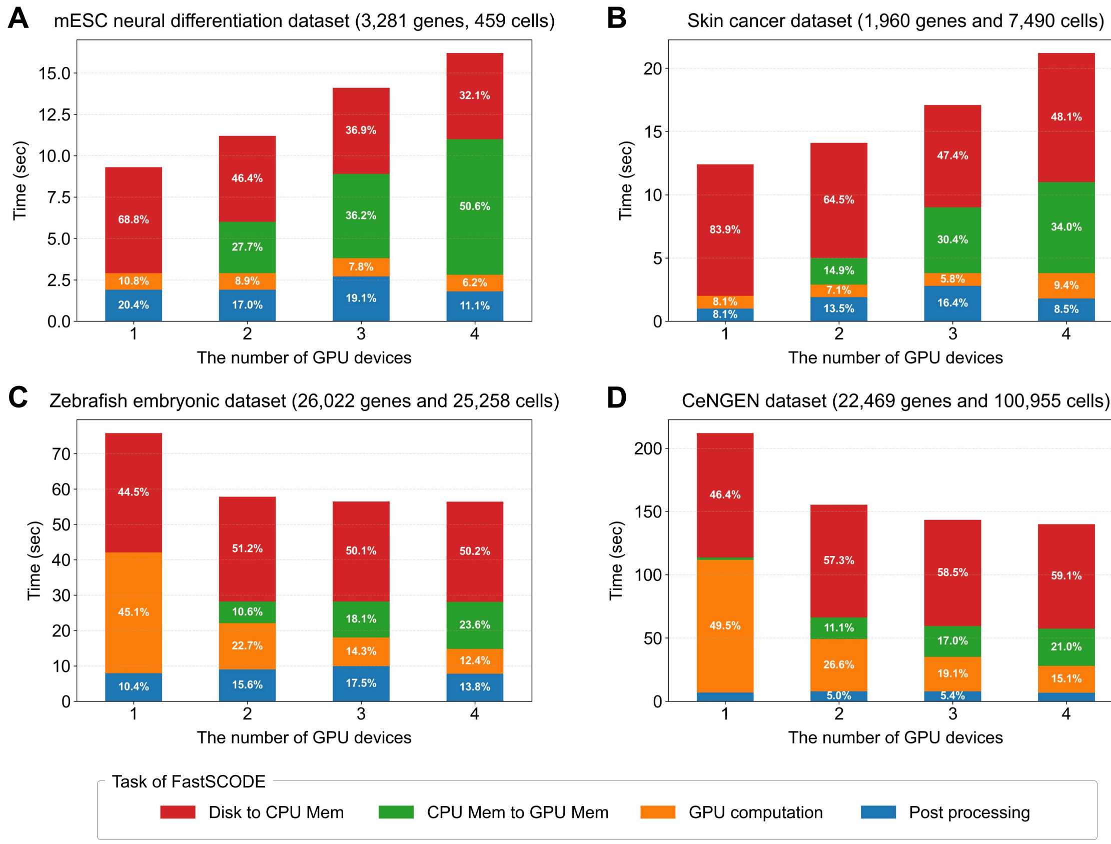

Figure S3. Comparison of runtime breakdown across the different number of GPU devices on the **(A)** mESC neural differentiation, **(B)** skin cancer, **(C)** zebrafish embryonic, and **(D)** CeNGEN datasets, evaluated on System 5 (NVIDIA RTX 4090 GPUs).

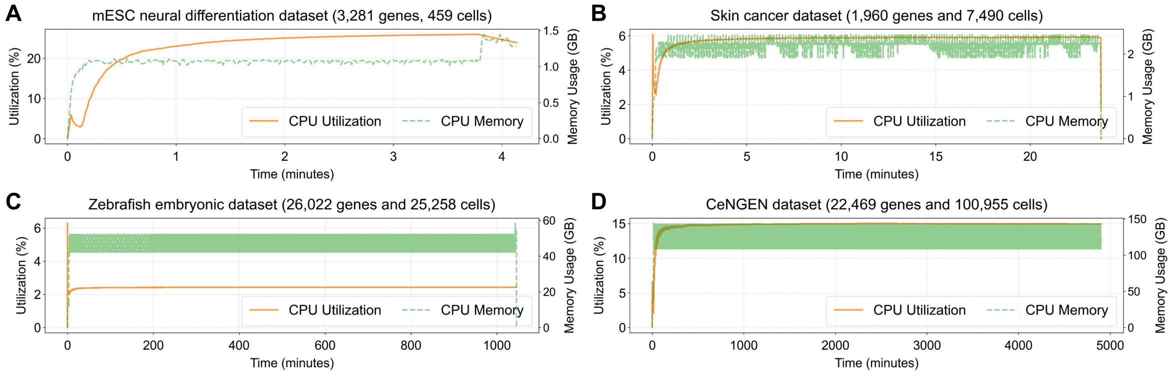

Figure S4. Resource utilization of the original SCODE for the **(A)** mESC neural differentiation, **(B)** skin cancer, **(C)** zebrafish embryonic, and **(D)** CeNGEN datasets, evaluated on System 1 (Intel Xeon Silver 4214R 2.4GHz CPU).

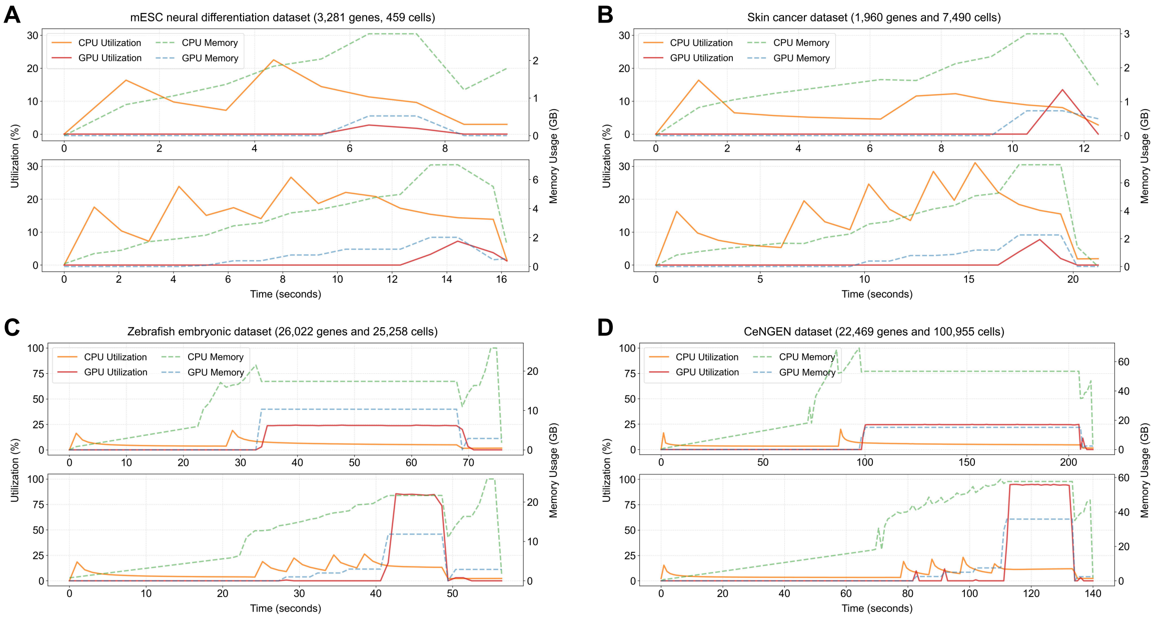

Figure S5. Resource utilization of FastSCODE under single-GPU (upper) and four-GPU (lower) conditions for the (A) mESC neural differentiation, (B) skin cancer, (C) zebrafish embryonic, and (D) CeNGEN datasets. CPU core, CPU memory, GPU core, and GPU memory utilization are measured on System 5 (NVIDIA RTX 4090 GPUs). A GPU utilization of 100% indicates that all cores of the four GPUs were fully occupied at maximum capacity. See also Table R1 for computing system configurations.

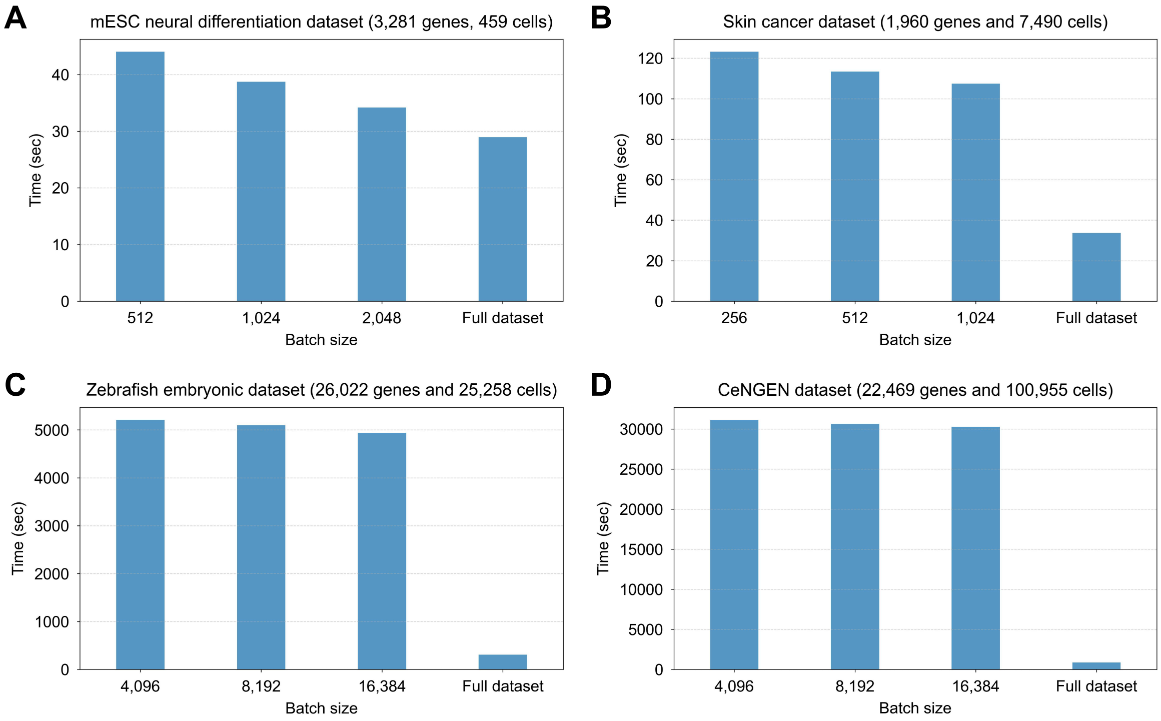

Figure S6. Impact of batch size on computational speed. Execution times of FastSCODE on the **(A)** mESC dataset, **(B)** skin cancer dataset, **(C)** zebrafish embryonic dataset, and **(D)** CeNGEN dataset, evaluated on System 5 (NVIDIA RTX 4090 GPUs).

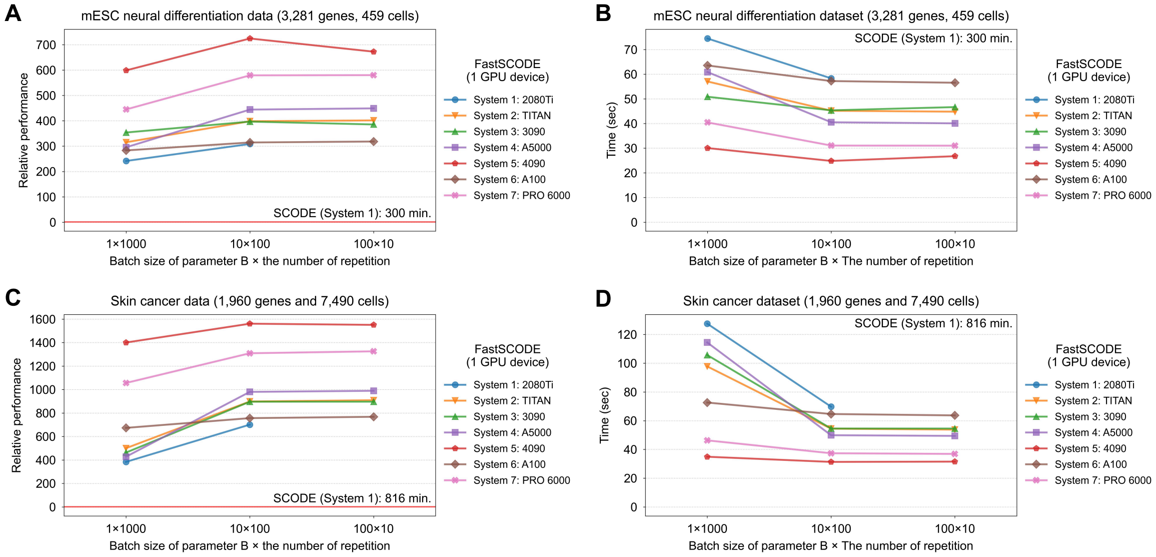

Figure S7. Impact of the batch size of parameter **B** on computational speed. Execution times and relative performance of FastSCODE on the **(A-B)** mESC dataset and **(C-D)** skin cancer dataset, respectively. System 1 (Intel Xeon Silver 4214R 2.4GHz CPU) was used for all experiments of the original SCODE.

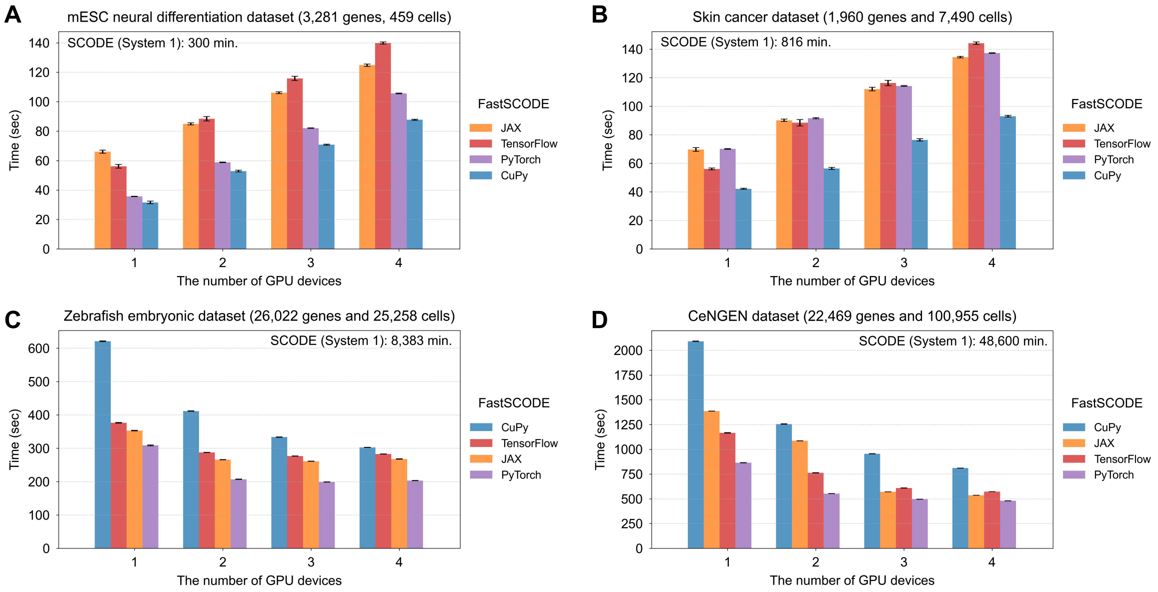

Figure S8. Execution times of FastSCODE across different manycore acceleration frameworks on the **(A)** mESC neural differentiation dataset, **(B)** skin cancer dataset, **(C)** zebrafish embryonic dataset, and **(D)** CeNGEN dataset, evaluated on System 5 (NVIDIA RTX 4090 GPUs). System 1 (Intel Xeon Silver 4214R 2.4 GHz CPU) was used for all experiments of the original SCODE.

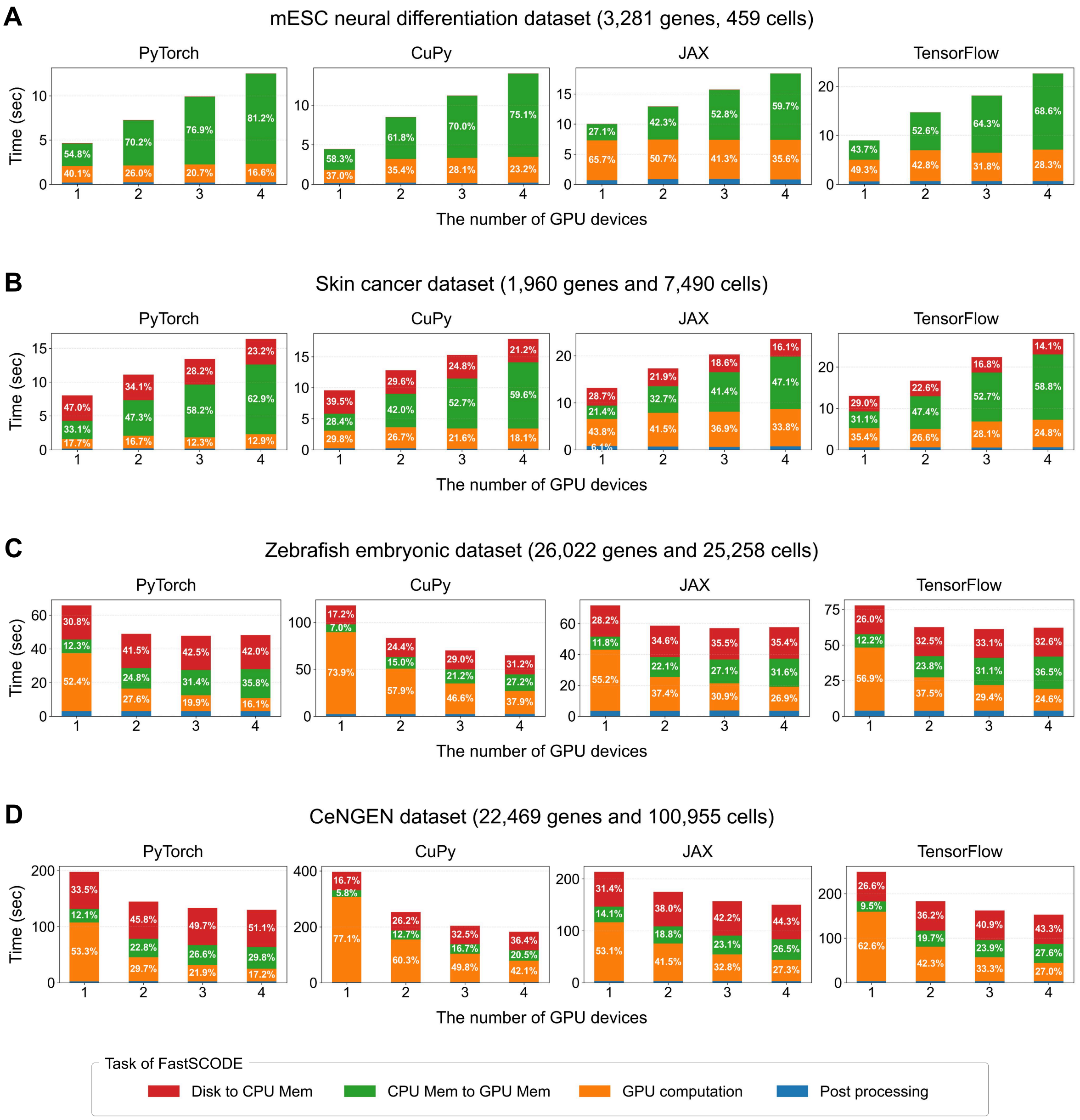

Figure S9. Comparison of runtime breakdown of four acceleration frameworks across the different number of GPU devices on the **(A)** mESC neural differentiation, **(B)** skin cancer, **(C)** zebrafish embryonic, and **(D)** CeNGEN datasets, evaluated on System 5 (NVIDIA RTX 4090 GPUs).

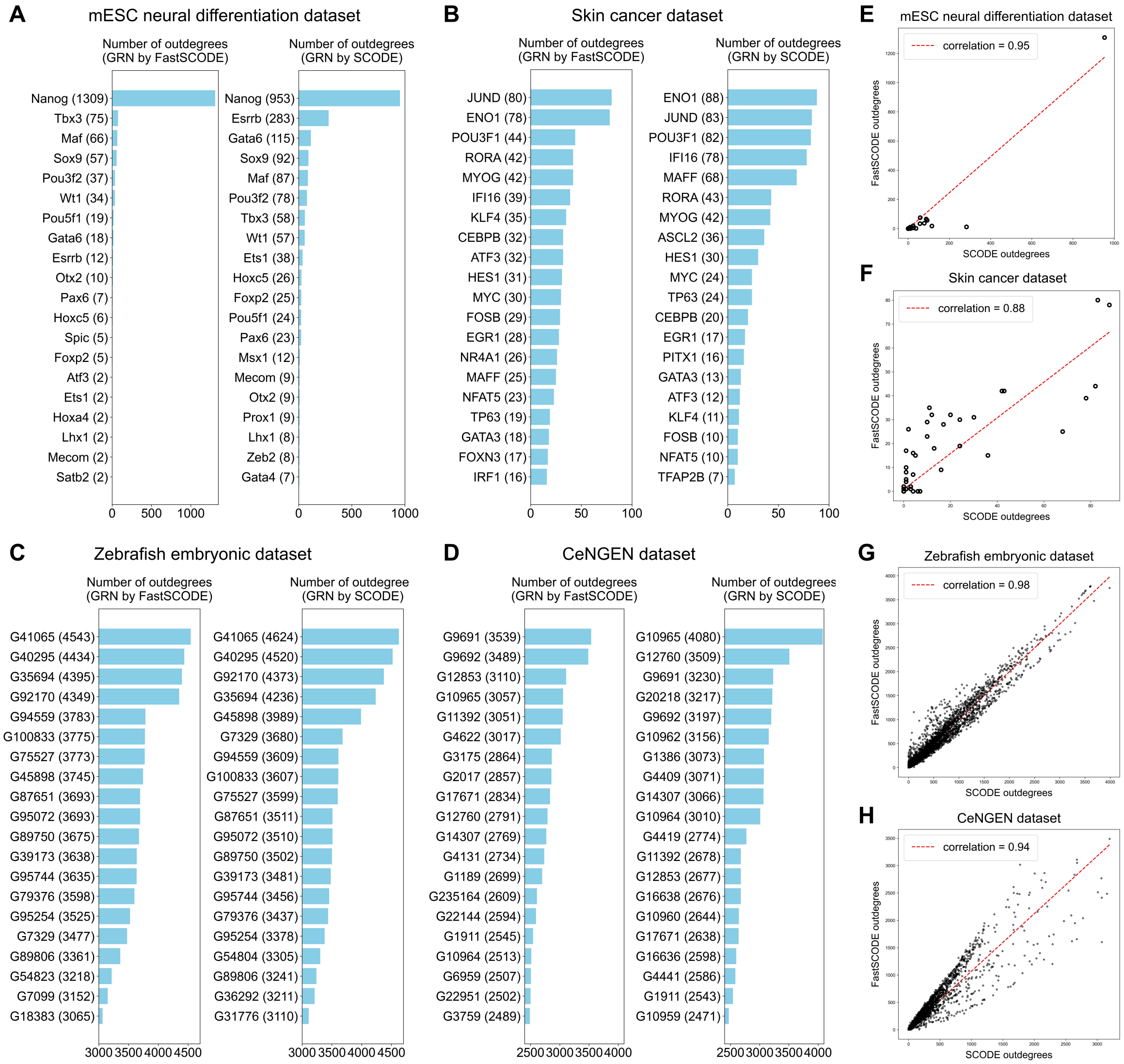

Figure S10. Comparison of consistency between FastSCODE and the original SCODE. The top outdegree gene lists of the GRN inferred by FastSCODE and SCODE are compared for the **(A)** mESC, **(B)** skin cancer, **(C)** zebrafish, and **(D)** CeNGEN datasets. In addition, the Pearson correlations of outdegree values between FastSCODE and SCODE are presented for the **(E)** mESC, **(F)** skin cancer, **(G)** zebrafish, and **(H)** CeNGEN datasets.
